# Supplementary material for: Evaluation of genetic variation and host resistance to wheat stem rust pathogen (Puccinia graminis f. sp. tritici) in bread wheat (Triticum aestivum L.) varieties grown in Türkiye
Source: PeerJ. 2024 Jun 25;12:e17633. doi: 10.7717/peerj.17633 (PMC11212643; doi:10.7717/peerj.17633)
Supplement: Supplemental Information 2 [file peerj-12-17633-s002.docx]

**SUPPLEMENTARY FIGURES**


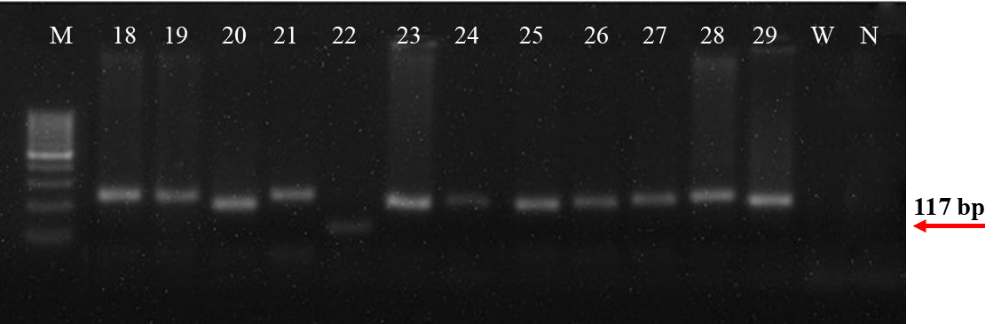


**Figure S1.** Gel electrophoretogram of wheat varieties amplified in PCR using WMC633 marker to the linked with the *Sr22* resistance gene. The red arrow shows the band size of *Sr22*-carrying (117 bp) and not carrying (171, 191 and 211 bp). W: Water, N: Negative control (Morocco), M: 100 bp DNA Ladder (Thermo Fisher Scientific, USA).


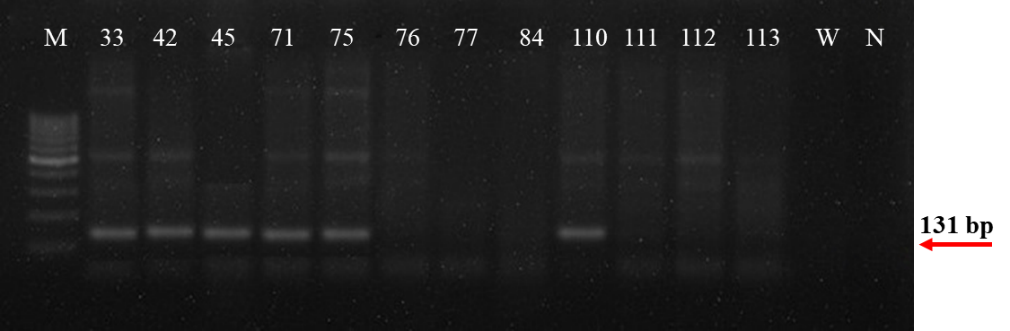


**Figure S2.** Gel electrophoretogram of wheat varieties amplified in PCR using GB marker to the linked with the *Sr25* resistance gene. The red arrow shows the band size of *Sr25*-carrying (131 bp). W: Water, N: Negative control (Morocco), M: 100 bp DNA Ladder (Thermo Fisher Scientific, USA).


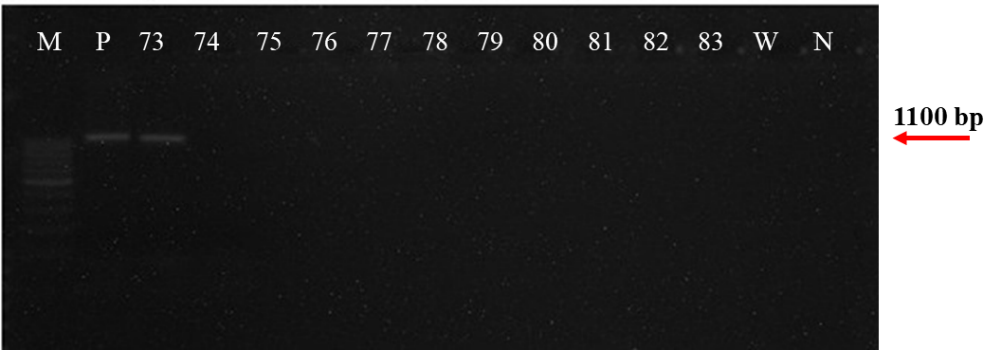


**Figure S3.** Gel electrophoretogram of wheat varieties amplified in PCR using Iag95 marker to the linked with the *Sr31* resistance gene. The red arrow shows the band size of *Sr31*-carrying (1100 bp). P: Positive control, W: Water, N: Negative control (Morocco), M: 100 bp DNA Ladder (Thermo Fisher Scientific, USA).


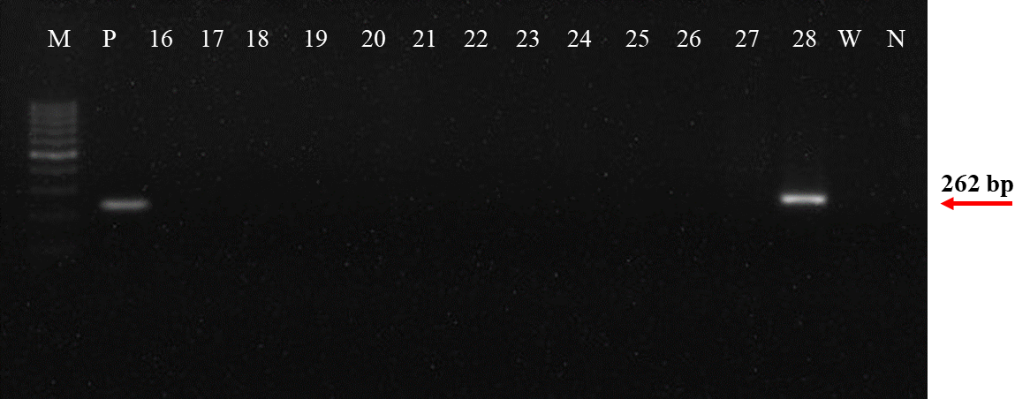


**Figure S4.** Gel electrophoretogram of wheat varieties amplified in PCR using primers to the Ventriup/LN2 locus linked with the *Sr38* resistance gene. The red arrow shows the band size of *Sr38*-carrying (262 bp). P: Positive control, W: Water, N: Negative control (Morocco), M: 100 bp DNA Ladder (Thermo Fisher Scientific, USA).


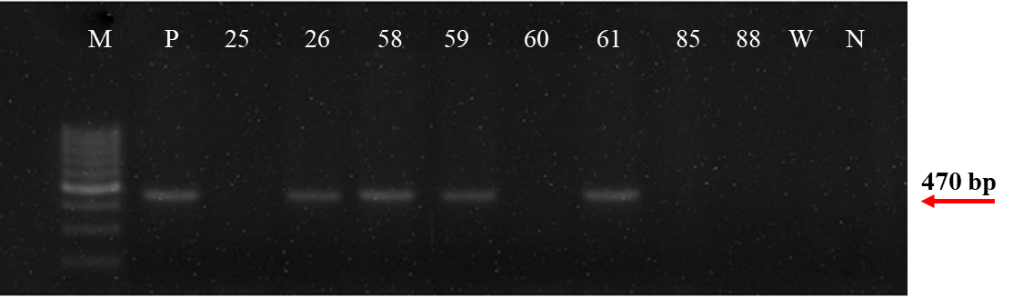


**Figure S5.** Gel electrophoretogram of wheat varieties amplified in PCR using *Sr50*-5p marker to the linked with the *Sr50* resistance gene. The red arrow shows the band size of *Sr50*-carrying (470 bp). P: Positive control, W: Water, N: Negative control (Morocco), M: 100 bp DNA Ladder (Thermo Fisher Scientific, USA).


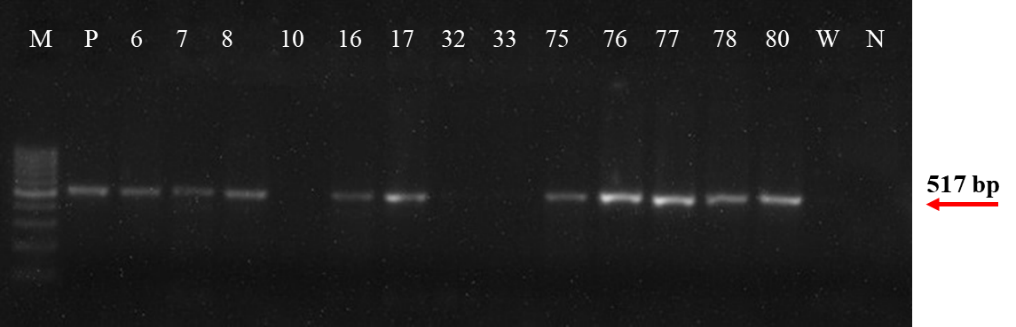


**Figure S6.** Gel electrophoretogram of wheat varieties amplified in PCR using L34DINT9F marker to the linked with the *Sr57* resistance gene. The red arrow shows the band size of *Sr57*-carrying (517 bp). P: Positive control, W: Water, N: Negative control (Morocco), M: 100 bp DNA Ladder (Thermo Fisher Scientific, USA).

**SUPPLEMENTARY TABLES**

**Table S1.** Name, release year and pedigree information associated with the 150 bread wheat varieties used in this study.

| **No** | **Cultivar name** | **Release year** | **Pedigree** | **No** | **Cultivar name** | **Release year** | **Pedigree** |
| --- | --- | --- | --- | --- | --- | --- | --- |
| 1 | Sivas 111/33 | 1937 | Pure-line selected from a landrace | 76 | Hanlı | 2007 | OK-82282//BOBWHITE/NEELKANT/3/F-4105-W |
| 2 | İkizce 96 | 1996 | ARTHUR*2/SIETE-CERROS-66//BRILL | 77 | Sakin | 2002 | PITIC-62(PI)/2*FUNO//VALDIVIA/3/CO-723595 |
| 3 | Mızrak | 1998 | POLYCROSS//C-126-15/C-47-6/3/YESILKOY-1978-79-7 | 78 | Özcan | 2004 | K-8/MM-2 |
| 4 | Uzunyayla | 1998 | HYSLOP/SIETE-CERROS-66//YAYLA-305/6/NADADORES-63/CO-652643/4/NAINARI-60/MAYO-54//NAINARI-60/KADAS/3/NS-220/5/HYSLOP/SIETE-CERROS-66 | 79 | Cumhuriyet 75 | 1976 | SONORA-64*2//TEZANOS-PINTOS-PRECOZ/YAQUI-54/3/ANDES-64-A/4/2*FROCOR//YAQUI/KENTANA |
| 5 | Bayraktar 2000 | 2000 | CHISHOLM(CSM)/GEREK-79 | 80 | Marmara 86 | 1986 | AVRORA//KALYANSONA/BLUEBIRD/3/(SIB)WOODPECKER |
| 6 | Atlı-2002 | 2002 | HYSLOP/SIETE-CERROS-66//SADOVO-1 | 81 | Kaklıç 88 | 1988 | KAVKAZ/(SIB)BUHO//KALYANSONA/BLUEBIRD |
| 7 | Zencirci-2002 | 2002 | STURDY/KIRAC-66 | 82 | Basri Bey 95 | 1995 | JUPATECO-73/(SIB)BLUEJAY//URES-81 |
| 8 | Eser | 2003 | AGRI/NACOZARI-76//LIRA | 83 | Kaşif Bey 95 | 1995 | HORK(SIB)/YAMHILL//KALYANSONA/BLUEBIRD” |
| 9 | Seval | 2004 | BOLAL-2973/NO64/3/ARTHUR*2/SIETE-CERROS-66//BOLAL-2973 | 84 | Gönen 98 | 1998 | II-8156-R/MARA//BLUEBIRD |
| 10 | Tosunbey | 2004 | ECVD-12/KIRAC-66//(SIB) CROW | 85 | Ziyabey 98 | 1998 | NORD-DESPREZ/VG-9144// KALYANSONA/BLUEBIRD/3/YACO/4/VEERY-5 |
| 11 | Kenanbey | 2009 | GEREK-79//CO-652643/KIRAC-66 | 86 | Meta 2002 | 2002 | NORD-DESPREZ/VG-9144//KALYANSONA/BLUEBIRD/3/YACO/4/VEERY-5 |
| 12 | Nis.22 | 1966 | Composite variety | 87 | Alibey | 2004 | KAUZ,MEX*2//SAPSUCKER/MONCHO/3/KAUZ,MEX |
| 13 | P 8-6 | 1966 | Ak702/Sertak52//Yayla305/Melez13 | 88 | Menemen | 2004 | JUPATECO-73/BLUEJAY//URES-81 |
| 14 | Melez 13 | 1944 | Composite variety | 89 | Çukurova 86 | 1986 | BLUEBIRD/KALYANSONA |
| 15 | Ak 702 | 1931 | Composite variety | 90 | Doğankent 1 | 1991 | FLICKER/HORK |
| 16 | Sertak 52 | 1936 | Composite variety | 91 | Seri 82 | 1991 | KAVKAZ/(SIB)BUHO//KALYANSONA/BLUEBIRD |
| 17 | Yayla 305 | 1939 | Composite variety | 92 | Seyhan 95 | 1995 | JUPATECO-73/(SIB)BLUEJAY//URES-81 |
| 18 | Porsuk-2800 | 1976 | N10B/3/27-15/RIO//REX-53/4/ BURT | 93 | Adana-99 | 1999 | PFAU/SERI-82//(SIB)BOBWHITE” |
| 19 | Gerek 79 | 1979 | MENTANA/MAYO-48//4-11/3/YAYLA-305 | 94 | Ceyhan-99 | 1999 | BLUEJAY(SIB)/COCORAQUE-75 |
| 20 | Atay-85 | 1985 | HYSLOP/SIETE-CERROS-66 | 95 | Pandas | 2001 | ORSO//BEZOSTAYA-1/S-1/3/ GENEROSO-7/CONTO-MARZOTTO |
| 21 | Kutluk 94 | 1994 | KRASNODARSKAYA//INIA/LILIFEN/3/CALIBASAN | 96 | Yüreğir-89 | 2002 | HD-1220/3*KALYANSONA// NACOZARI-76 |
| 22 | Kırgız 95 | 1995 | DOMANIC/AVRORA | 97 | Karatopak | 2006 | TESIA-79/VEERY(SIB)// SERI-82 |
| 23 | Sultan 95 | 1995 | AGRI/NACOZARI-76 | 98 | Osmaniyem | 2006 | TUJ/ONELTO |
| 24 | Süzen 97 | 1997 | C-126-15/COLLAFEN/3/NORIN-10-BREVOR/P-14//P-101/4/(KRC)KIRAC-66 | 99 | Altın Başak | 2013 | CHEN/TR.TA//BACANORA-88/3/2*KAUZ |
| 25 | Yıldız 98 | 1998 | SEL.55-1744/P-101//MAYA-74/3/MUSALA/(PRM)PRIMO//MAYA-74/(SIB)ALONDRA | 100 | Gökkan | 2013 | KRICHAUFF/FINSI |
| 26 | Harmankaya-99 | 1999 | FUNDULEA-29/2*LOVRIN-32 | 101 | Seri 2013 | 2013 | WEAVER/4/NACOZARI- 76/THATCHER/AC//3*PAVON- 76/3/MIRLO/BUCKBUCK |
| 27 | Altay 2000 | 2000 | ES-14//YEKTAY/BLUEBOY-2 | 102 | Yakamoz | 2014 | BL-1496/MILAN/3/CROC-1/(205)TR.TA//KAUZ |
| 28 | Çetinel 2000 | 2000 | MALCOLM/4/VPM 1/MOISSON 951//HILL 81/3/STEPHENS | 103 | Gemini | 1987 | AUTONOMIA//AUTONOMIA/AQUILA |
| 29 | Alpu 2001 | 2001 | ID800994.W/VEE | 104 | Köksal-2000 | 2001 | Unknown |
| 30 | Sönmez 2001 | 2001 | BEZ//BEZ/TVR/3/KREMENA/LOV29/4/KATIA1 | 105 | Genç 88 | 1988 | CIANO-67(SIB)/ NACOZARI-76// CORRECAMINOS(CC)/ INIA-66/3/BLUEBIRD/ NARINO-59 |
| 31 | Soyer02 | 2002 | ATAY-85/GALVEZ-S-87 | 106 | Özkan | 2011 | VORONA/CIANO-79//KAUZ |
| 32 | Müfitbey | 2006 | NGDA146/4/YMH/TOB//MCD/ 3/LIRA/5/F130L1.12 | 107 | Carisma | 2011 | D-29/F-65 |
| 33 | Nacibey | 2008 | F900K/3/EGL//BUC/PVN | 108 | Esperia | 2011 | B-16-3/LINEA-RUSSA |
| 34 | ES 26 | 2010 | LLKOFEN/GEREK79*4 | 109 | Sagittario | 2001 | ADAM/Z-282 |
| 35 | Yunus | 2012 | SG-S1915/FANDANGO | 110 | Bone de | 2013 | Unknown |
| 36 | Mesut | 2013 | MV-8/5/BEZOSTAYA-1//BEZOSTAYA-1/TEVERE/3/KREMENA/LOVRIN-29/4/KATYA-1 | 111 | Bora | 2014 | H-31/TRAP-1-F-2//ENESCO |
| 37 | Kınacı-97 | 1997 | YAMHILL/TOBARI-66//MCDERMID/3/LIRA | 112 | Genesi | 2014 | COLFIORITO/HEREWARD |
| 38 | Karahan-99 | 1999 | C-126-15/COLLAFEN/3/NORIN-10-BREVOR/P-14//(P101)PULLMAN-101/4/KIRAC-66 | 113 | Syrena odes'ka | 2008 | Unknown |
| 39 | Bağcı-2002 | 2002 | HN7/OROFEN//BEIJING 8/3/SERI M82/4/74CB462/TRAPPER//VONA | 114 | Nota | 2013 | LUTESCENS-2618-G-26465/LUTESCENS-5056-h-44-3//LUTESCENS-5056-h-44-3 |
| 40 | Konya-2002 | 2002 | KANRED/ TENMARQ//P-211-6/3/2183/ CO-652643/LANCER | 115 | Yubileynaya | 2013 | Unknown |
| 41 | Ahmetağa | 2004 | Unknown | 116 | Galil | 2002 | HORK/YAMHILL//KALYANSONA/BLUEBIRD/3/BOBWHİTE ‘S’ |
| 42 | Ekiz | 2004 | F-885-K-1-1/SIOUXLAND | 117 | Özdemirbey-97 | 2003 | JUPATECO-73/(SIB)BLUEJAY//URES-81 |
| 43 | Eraybey | 2012 | F-10-S-1/CHISHOLM | 118 | Pinzon | 2011 | Unknown |
| 44 | Kırkpınar 79 | 1979 | HYSLOP/SIETE-CERROS-66 | 119 | Flamura 85 | 1999 | RANNYAYA-12/NADADORES-63//LOVRIN-12 |
| 45 | Murat-1 | 1991 | Unknown | 120 | Alka | 2011 | HANA/MERCIA |
| 46 | Kate A-1 | 1988 | KHEBROS/BEZOSTAJA-1 | 121 | Guadalupe | 2007 | 165613/RECITAL |
| 47 | Pehlivan | 1998 | BEZOSTAYA-1/TEVERE/5/CENTRIFEN/BEZOSTAYA-1//SUWEON-92/CI-13645/3/NAINARI-60/4/(SIB)EMU | 122 | Tina | 2005 | SANA/GALA |
| 48 | Prostor | 1999 | RUSALKA-PODOBRENA/NADADORES-63 | 123 | Tosun 144 | 1975 | BEZOSTAYA-1/54-T-72 |
| 49 | Saroz 95 | 1999 | COR-71-11460/3/PKG/LOVRIN-13//JINGSWON-3 | 124 | Krasunia odes'ka | 2008 | Unknown |
| 50 | Atilla-12 | 2001 | MIRONOVSKAYA-808/BEZOSTAYA-1//BEZOSTAYA- 1/3/BEZOSTAYA-1/PRODUTTORE//BEZOSTAYA-1 | 125 | Tanya | 2012 | GRADO(TR.CE)/2*SKIFYANKA |
| 51 | Saraybosna | 2001 | OSJECKA-20/OSK-4.216-2-76 | 126 | Dariel | 2002 | HORK/YAMHILL//KALYANSONA/BLUEBIRD |
| 52 | Gelibolu | 2005 | 506/88-113 | 127 | Bancal | 2006 | Unknown |
| 53 | Tekirdağ | 2005 | Unknown | 128 | Nina | 2005 | SANA(ZG-213-82)/GALA |
| 54 | Aldane | 2009 | BUL-2477-2/3/093-44/AVRORA//BEZOSTAYA-1 | 129 | Mihelca | 2014 | ZG-1325-78/SO-1065 |
| 55 | Selimiye | 2009 | LAU/AGD/3/ODESSKAYA-95//OLVIYA/B16 | 130 | Prima | 2014 | SANA/GALA |
| 56 | Bereket | 2010 | KIRAC-66/BEZOSTAYA-1//SUP/3/WEIQUE-RED-MACE(WRM)/2*HUACAMAYO/4/DONS/5/KATE/MVM/6/PRES | 131 | Segor | 2013 | Unknown |
| 57 | Saban | 2014 | TRAKIYA/3/MV-C-410-90/GK-KALAKA//MV-C-410-90/FTM-11 | 132 | Colfiorito | 2010 | IRNERIO/IS-237-T//ARGELATO |
| 58 | Lancer | 1977 | BEZOSTAYA-1/4/II-50-72//YAQUI-54/N10B/3/MARNE- DESPREZ(MD)/IGA-BORDEAUX | 133 | Mane Nick | 2012 | Unknown |
| 59 | Doğu 88 | 1990 | BEZOSTAYA-1/DANNE// CO-725052 | 134 | Vittorio | 2011 | LANCOTA/KALYANSONA//JE-4-B |
| 60 | Karasu 90 | 1990 | LOVRIN-11/BOLAL-2973//MIRONOVSKAYA-264 | 135 | Quality | 2012 | Unknown |
| 61 | Palandöken 97 | 1997 | AVRORA//YAKTANA-54*2/NORIN-10-BREVOR/3/II-8260/5/PONCA(PNC)/CM//NB- 6977/3/CC/LNIA//BLUEBIRD/4/MEXIPAK/IKR/FUNO | 136 | Adagio | 2013 | Unknown |
| 62 | Alparslan | 2001 | TX-69-A-509-2//BBY2/FOX | 137 | Iridium | 2012 | ORATORIO/SHANGO |
| 63 | Nenehatun | 2001 | NORD DEPREZ/PULLMAN SELECTION 101//BLUEBOY | 138 | Geya I | 2011 | Unknown |
| 64 | Daphan | 2002 | JUPATECO-73/4/COLLAFEN/3/II-14.53/ODIN,SWE//VOGEL-1(CI-14431)/WA-00477 | 139 | Anapo | 2011 | EG-52/BEL-118 |
| 65 | Ayyıldız | 2011 | NONGDA-146/4/YAMHILL/TOBARI-66//MCDERMID/3/LIRA/5/F-130-L-1-12 | 140 | Rumeli | 2012 | Unknown |
| 66 | Kırik | 2010 | Unknown | 141 | Turkuaz | 2012 | Unknown |
| 67 | Karacadağ 98 | 1998 | RED-RIVER-68/WW-15/3/ BAJIO/2*OLESEN// BONANZA/4/NACOZARI-76 | 142 | Tekira | 2009 | Unknown |
| 68 | Nurkent | 2001 | HD-1220/3*KALYANSONA// NACOZARI-76 | 143 | Nogal | 2012 | Unknown |
| 69 | Tekin | 2014 | WEEBILL-1*2/TUKURU | 144 | Adelaide | 2013 | Unknown |
| 70 | İnia 66 | 1970 | LERMA-ROJO-64/SONORA-64 | 145 | Antille | 2013 | Unknown |
| 71 | Bezostaja-1 | 1968 | LUTESCENS-17,UKR/ SKOROSPELKA-2 | 146 | Avorio | 2013 | Unknown |
| 72 | Bandırma 97 | 1997 | BOBWHITE/PARULA | 147 | MV Suba | 2012 | ERYTHROSPERMUM-1778-87/2*MV-MAGDALENA |
| 73 | Karacabey 97 | 1997 | VEERY-5/PAVON-76/3/ GOLDEN-VALLEY/ AZTECA-67//MUSALA | 148 | Aglika | 2012 | GP-2558-128/PLISKA |
| 74 | Momtchill | 2000 | NS-11-33/AVRORA | 149 | İnci20 | 2012 | Unknown |
| 75 | Beşköprü | 2007 | 362-K-2-111/6/NEELKANT/5/TOBARI-66/CIANO-67//TOBARI-66/8156/3/CALIDAD//BLUEBIRD/CIANO-67/4/TORIM-73 | 150 | Hawk (Şahin) | - | - |

**Table S2.** Host reactions (HR) and coefficient of infection (CI) value of 150 bread wheat varieties at adult-plant stages in natural infection in 2020 and 2021 growing seasons and molecular screening results of eight stem rust resistance genes.

|  | | **Resistance genes** | | | | | | | | **Resistance at adult plant** | | | |
| --- | --- | --- | --- | --- | --- | --- | --- | --- | --- | --- | --- | --- | --- |
|  |  |  |  |  |  |  |  |  |  | **2020** | | **2021** | |
| **No** | **Variety** | ***Sr22*** | ***Sr24*** | ***Sr25*** | ***Sr26*** | ***Sr31*** | ***Sr38*** | ***Sr50*** | ***Sr57*** | **HR** | **CI** | **HR** | **CI** |
| 1 | Sivas 111/33 | - | - | - | - | - | - | - | + | 10R | 2 | 20R | 4 |
| 2 | İkizce 96 | - | - | - | - | - | - | - | - | 10MR | 4 | 30MR | 12 |
| 3 | Mızrak | - | - | - | - | - | - | - | - | 10R | 2 | 20MR | 8 |
| 4 | Uzunyayla | - | - | - | - | - | - | - | - | 70S | 70 | 70S | 70 |
| 5 | Bayraktar 2000 | - | - | - | - | - | - | - | - | 90S | 90 | 70S | 60 |
| 6 | Atlı-2002 | - | - | - | - | - | - | - | + | 5R | 1 | 10R | 2 |
| 7 | Zencirci-2002 | - | - | - | - | - | - | - | + | 10R | 2 | 20R | 4 |
| 8 | Eser | - | - | - | - | - | - | - | + | 40MS | 32 | 30MS | 24 |
| 9 | Seval | - | - | - | - | - | - | - | + | 10R | 2 | 20MR | 8 |
| 10 | Tosunbey | - | - | - | - | - | - | - | - | 5R | 1 | 20MR | 8 |
| 11 | Kenanbey | - | - | - | - | - | - | - | + | 5R | 1 | 10MR | 4 |
| 12 | 4-22 | - | - | - | - | - | - | - | - | 80S | 80 | 70S | 70 |
| 13 | P 8-6 | - | - | - | - | - | - | - | - | 100S | 100 | 90S | 90 |
| 14 | Melez | - | - | - | - | - | - | - | - | 20MR | 8 | 50MS | 40 |
| 15 | Ak 702 | - | - | - | - | - | - | - | + | 40MS | 32 | 30MS | 24 |
| 16 | Sertak | - | - | - | - | - | - | - | + | 30MS | 24 | 30MS | 24 |
| 17 | Yayla 305 | - | - | - | - | - | - | - | + | 10MR | 4 | 20MR | 8 |
| 18 | Porsuk-2800 | - | - | - | - | - | - | - | - | 30MS | 24 | 50MS | 40 |
| 19 | Gerek 79 | - | - | - | - | - | - | - | - | 40MS | 32 | 50MS | 40 |
| 20 | Atay-85 | - | - | - | - | - | - | - | - | 10MR | 4 | 30MR | 12 |
| 21 | Kutluk 94 | - | - | - | - | - | - | - | - | 10MR | 4 | 20MR | 8 |
| 22 | Kırgız 95 | + | - | - | - | - | - | - | - | 5R | 1 | 10R | 2 |
| 23 | Sultan 95 | - | - | - | - | - | - | - | + | 5R | 1 | 20R | 4 |
| 24 | Süzen 97 | - | - | - | - | - | - | - | - | 70S | 70 | 60S | 60 |
| 25 | Yıldız 98 | - | - | - | - | - | - | - | + | 20MS | 16 | 30MS | 24 |
| 26 | Harmankaya-99 | - | - | - | - | - | - | + | - | 10MR | 4 | 10MR | 4 |
| 27 | Altay 2000 | - | - | - | - | - | - | - | - | 20MR | 8 | 30MS | 24 |
| 28 | Çetinel 2000 | - | - | - | - | - | + | - | + | 20MR | 8 | 10MR | 4 |
| 29 | Alpu 2001 | - | - | - | - | - | - | - | + | 20MS | 16 | 20MS | 16 |
| 30 | Sönmez 2001 | - | - | - | - | - | - | - | + | 5R | 1 | 20R | 4 |
| 31 | Soyer02 | - | - | - | - | - | - | - | + | 10MR | 4 | 20MR | 8 |
| 32 | Müfitbey | - | - | - | - | - | - | - | - | 10MR | 4 | 20MR | 8 |
| 33 | Nacibey | - | - | + | - | - | - | - | - | 5R | 1 | 10R | 2 |
| 34 | ES 26 | - | - | - | - | - | - | - | - | 10MR | 4 | 30MR | 12 |
| 35 | Yunus | - | - | - | - | - | - | - | + | 40MS | 32 | 50MS | 40 |
| 36 | Mesut | - | - | - | - | - | - | - | + | 5R | 1 | 20MR | 8 |
| 37 | Kınacı-97 | - | - | - | - | - | - | - | - | 10R | 2 | 40MR | 16 |
| 38 | Karahan-99 | - | - | - | - | - | - | - | + | 60S | 60 | 50MS | 40 |
| 39 | Bağcı-2002 | - | - | - | - | - | - | - | - | 60S | 60 | 70S | 70 |
| 40 | Konya-2002 | - | - | - | - | - | - | - | - | 30MS | 24 | 60MS | 48 |
| 41 | Ahmetağa | - | - | - | - | - | - | - | - | 60MS | 48 | 70S | 70 |
| 42 | Ekiz | - | - | + | - | - | - | - | + | 20R | 4 | 10R | 2 |
| 43 | Eraybey | - | - | - | - | - | - | - | + | 5R | 1 | 20R | 4 |
| 44 | Kırkpınar 79 | - | - | - | - | - | - | - | + | 20MR | 8 | 10MR | 4 |
| 45 | Murat-1 | - | - | + | - | - | - | - | - | 5R | 1 | 10R | 2 |
| 46 | Kate A-1 | - | - | - | - | - | - | - | + | 50MS | 40 | 60MS | 48 |
| 47 | Pehlivan | - | - | - | - | - | - | - | + | 30MS | 24 | 50MS | 40 |
| 48 | Prostor | - | - | - | - | - | - | - | + | 10R | 2 | 20R | 4 |
| 49 | Saroz 95 | - | - | - | - | - | - | - | + | 90S | 90 | 80S | 80 |
| 50 | Atilla-12 | - | - | - | - | - | - | - | + | 90S | 90 | 70S | 70 |
| 51 | Saraybosna | - | - | - | - | - | - | - | + | 90S | 90 | 70S | 70 |
| 52 | Gelibolu | - | - | - | - | - | - | - | + | 10R | 2 | 20R | 4 |
| 53 | Tekirdağ | - | - | - | - | - | - | - | - | 10R | 2 | 20MR | 8 |
| 54 | Aldane | - | - | - | - | - | - | - | - | 10R | 2 | 10MR | 4 |
| 55 | Selimiye | - | - | - | - | - | - | - | + | 5MR | 2 | 10MR | 4 |
| 56 | Bereket | - | - | - | - | - | - | - | - | 50S | 50 | 70S | 70 |
| 57 | Saban | - | - | - | - | - | - | - | + | 10R | 2 | 20R | 4 |
| 58 | Lancer | - | - | - | - | - | - | + | - | 10R | 2 | 10R | 2 |
| 59 | Doğu 88 | - | - | - | - | - | - | + | + | 70S | 70 | 70S | 70 |
| 60 | Karasu 90 | - | - | - | - | - | - | - | + | 80S | 80 | 70S | 70 |
| 61 | Palandöken 97 | - | - | - | - | - | - | + | - | 80S | 80 | 70S | 70 |
| 62 | Alparslan | - | - | - | - | - | - | - | - | 70S | 70 | 70S | 70 |
| 63 | Nenehatun | - | - | - | - | - | - | - | - | 80S | 80 | 70S | 70 |
| 64 | Daphan | - | - | - | - | - | - | - | + | 10MS | 8 | 20MS | 16 |
| 65 | Ayyıldız | - | - | - | - | - | - | - | + | 5R | 1 | 10R | 2 |
| 66 | Kırik | - | - | - | - | - | - | - | - | 100S | 100 | 80S | 80 |
| 67 | Karacadağ 98 | - | - | - | - | - | - | - | - | 70S | 70 | 70S | 70 |
| 68 | Nurkent | - | - | - | - | - | - | - | + | 20MS | 16 | 20MS | 16 |
| 69 | Tekin | - | - | - | - | - | - | - | + | 5R | 1 | 10R | 2 |
| 70 | İnia 66 | - | - | - | - | - | - | - | + | 10MR | 4 | 20MR | 8 |
| 71 | Bezostaja-1 | - | - | + | - | - | - | - | + | 5R | 1 | 10R | 2 |
| 72 | Bandırma 97 | - | - | - | - | - | - | - | + | 0 | 0 | 0 | 0 |
| 73 | Karacabey 97 | - | - | - | - | + | - | - | + | 10MR | 4 | 10MR | 4 |
| 74 | Momtchill | - | - | - | - | - | - | - | + | 5R | 1 | 10MR | 4 |
| 75 | Beşköprü | - | - | + | - | - | - | - | + | 10MR | 4 | 20MR | 8 |
| 76 | Hanlı | - | - | - | - | - | - | - | + | 10R | 2 | 20R | 4 |
| 77 | Sakin | - | - | - | - | - | - | - | + | 10MR | 4 | 20MR | 8 |
| 78 | Özcan | - | - | - | - | - | - | - | + | 5R | 1 | 10MR | 4 |
| 79 | Cumhuriyet 75 | - | - | - | - | - | - | - | - | 30MR | 12 | 50MS | 40 |
| 80 | Marmara 86 | - | - | - | - | - | - | - | + | 0 | 0 | 0 | 0 |
| 81 | Kaklıç 88 | - | - | - | - | - | - | - | + | 10MS | 8 | 30MS | 24 |
| 82 | Basri Bey 95 | - | - | - | - | - | - | - | + | 10MR | 4 | 20MR | 8 |
| 83 | Kaşif Bey 95 | - | - | - | - | - | - | - | + | 10MR | 4 | 5R | 1 |
| 84 | Gönen 98 | - | - | - | - | - | - | - | + | 5R | 1 | 20MR | 8 |
| 85 | Ziyabey 98 | - | - | - | - | - | - | - | - | 60MS | 48 | 50MS | 40 |
| 86 | Meta 2002 | - | - | - | - | - | - | + | + | 0 | 0 | 0 | 0 |
| 87 | Alibey | - | - | - | - | - | - | - | + | 10MS | 8 | 30MS | 24 |
| 88 | Menemen | - | - | - | - | - | - | - | + | 10MS | 8 | 40MS | 32 |
| 89 | Çukurova 86 | - | - | - | - | - | - | - | + | 20MS | 16 | 20MS | 16 |
| 90 | Doğankent 1 | - | - | - | - | - | - | - | + | 70S | 70 | 60S | 60 |
| 91 | Seri 82 | - | - | - | - | - | - | - | + | 20MS | 16 | 60S | 60 |
| 92 | Seyhan 95 | - | - | - | - | - | - | - | - | 70S | 70 | 70S | 70 |
| 93 | Adana-99 | - | - | - | - | - | - | - | + | 80MS | 64 | 50MS | 40 |
| 94 | Ceyhan-99 | - | - | - | - | - | - | - | + | 80MS | 64 | 50MS | 40 |
| 95 | Pandas (Panda) | - | - | - | - | - | - | - | - | 80S | 80 | 70S | 70 |
| 96 | Yüreğir-89 | - | - | - | - | - | - | + | - | 50MS | 40 | 40MS | 32 |
| 97 | Karatopak | - | - | - | - | - | - | - | + | 10R | 2 | 20R | 4 |
| 98 | Osmaniyem | - | - | - | - | - | - | - | + | 10MR | 4 | 20MR | 8 |
| 99 | Altın Başak | - | - | - | - | - | - | - | + | 5R | 1 | 10MR | 4 |
| 100 | Gökkan | - | - | - | - | - | - | - | + | 5R | 1 | 20MR | 8 |
| 101 | Seri 2013 | - | - | - | - | - | - | - | + | 10R | 2 | 10MR | 4 |
| 102 | Yakamoz | - | - | - | - | - | - | - | - | 50S | 50 | 60S | 60 |
| 103 | Gemini | - | - | - | - | - | - | - | + | 5R | 1 | 10R | 2 |
| 104 | Köksal-2000 | - | - | - | - | - | - | - | + | 20MR | 8 | 30MS | 24 |
| 105 | Genç 88 | - | - | - | - | - | - | - | + | 0 | 0 | 0 | 0 |
| 106 | Özkan | - | - | - | - | - | - | + | + | 30MR | 12 | 20MS | 16 |
| 107 | Carisma | - | - | - | - | - | - | - | + | 5R | 1 | 20R | 4 |
| 108 | Esperia | - | - | - | - | - | - | - | - | 30MS | 24 | 50MS | 40 |
| 109 | Sagittario | - | - | - | - | - | - | - | - | 40MS | 32 | 50S | 50 |
| 110 | Bone de | - | - | + | - | - | - | - | - | 10R | 2 | 10R | 2 |
| 111 | Bora | - | - | - | - | - | - | - | + | 0 | 0 | 0 | 0 |
| 112 | Genesi | - | - | - | - | - | - | - | + | 20MR | 8 | 20MR | 8 |
| 113 | Syrena odes'ka | - | - | - | - | - | - | - | + | 10R | 2 | 20MR | 8 |
| 114 | Nota | - | - | - | - | - | - | - | + | 5R | 1 | 10MR | 4 |
| 115 | Yubileynaya | - | - | - | - | - | - | - | + | 10MR | 4 | 20MR | 8 |
| 116 | Galil | - | - | - | - | - | - | - | + | 0 | 0 | 0 | 0 |
| 117 | Özdemirbey-97 | - | - | - | - | - | - | - | + | 10MR | 4 | 10MR | 4 |
| 118 | Pinzon | - | - | - | - | - | - | - | + | 0 | 0 | 0 | 0 |
| 119 | Flamura 85 | - | - | - | - | - | - | - | + | 0 | 0 | 0 | 0 |
| 120 | Alka | - | - | - | - | - | - | + | - | 0 | 0 | 0 | 0 |
| 121 | Guadalupe | - | - | - | - | - | - | - | + | 20MR | 8 | 10MR | 4 |
| 122 | Tina | - | - | - | - | - | - | - | + | 30MS | 24 | 20MR | 8 |
| 123 | Tosun 144 | - | - | - | - | - | - | - | + | 5R | 1 | 10MR | 4 |
| 124 | Krasunia odes'ka | - | - | - | - | - | - | - | + | 20MR | 8 | 30MR | 12 |
| 125 | Tanya | - | - | - | - | - | - | - | + | 5MR | 2 | 20MR | 8 |
| 126 | Dariel | - | - | - | - | - | - | - | + | 10MR | 4 | 20MS | 16 |
| 127 | Bancal | - | - | - | - | - | - | - | + | 0 | 0 | 0 | 0 |
| 128 | Nina | - | - | - | - | - | - | - | + | 20MR | 8 | 20MS | 16 |
| 129 | Mihelca | - | - | - | - | - | - | - | + | 10MR | 4 | 20MR | 8 |
| 130 | Prima | - | - | - | - | - | - | - | + | 40MR | 16 | 30MS | 24 |
| 131 | Segor | - | - | - | - | - | - | - | + | 20MR | 8 | 20MR | 8 |
| 132 | Colfiorito | - | - | - | - | - | - | - | + | 10MR | 4 | 30MR | 12 |
| 133 | Mane Nick | - | - | - | - | - | - | - | + | 5R | 1 | 5R | 1 |
| 134 | Vittorio | - | - | - | - | - | - | - | + | 30MS | 24 | 40MR | 16 |
| 135 | Quality | - | - | - | - | - | - | - | + | 30MR | 12 | 40MS | 32 |
| 136 | Adagio | - | - | - | - | - | - | - | + | 20MR | 8 | 20MR | 8 |
| 137 | Iridium | - | - | - | - | - | - | + | + | 10MR | 4 | 10MR | 4 |
| 138 | Geya I | - | - | - | - | - | - | - | + | 10MR | 4 | 20MR | 8 |
| 139 | Anapo | - | - | - | - | - | - | - | + | 0 | 0 | 0 | 0 |
| 140 | Rumeli | - | - | - | - | - | - | - | + | 5MR | 2 | 10MR | 4 |
| 141 | Turkuaz | - | - | - | - | - | - | - | + | 10MR | 4 | 20MR | 8 |
| 142 | Tekira | - | - | - | - | - | - | - | + | 0 | 0 | 0 | 0 |
| 143 | Nogal | - | - | - | - | - | - | - | + | 10MR | 4 | 10MR | 4 |
| 144 | Adelaide | - | - | - | - | - | - | - | - | 60S | 60 | 60S | 60 |
| 145 | Antille | - | - | - | - | - | - | - | - | 60S | 60 | 60S | 60 |
| 146 | Avorio | - | - | - | - | - | - | - | + | 10MR | 4 | 20MR | 8 |
| 147 | MV Suba | - | - | - | - | - | - | - | + | 5MR | 2 | 20MR | 8 |
| 148 | Aglika | - | - | - | - | - | - | - | + | 10MR | 4 | 20MS | 16 |
| 149 | İnci20 | - | - | - | - | - | - | - | - | 60S | 60 | 70S | 70 |
| 150 | Hawk (Şahin) | - | - | - | - | - | - | - | - | 50MS | 40 | 60S | 60 |
| 151 | Morocco | - | - | - | - | - | - | - | - | 80S | 80 | 80S | 80 |
